# Analysis of Reddit Discussions on Motivational Factors for Physical Activity: Cross-Sectional Study

**DOI:** 10.2196/54489

**Published:** 2025-01-13

**Authors:** Michal Shmueli-Scheuer, Yedidya Silverman, Israel Halperin, Yftach Gepner

**Affiliations:** 1 Department of Health Promotion School of Public Health, Faculty of Medical and Health Sciences Tel Aviv University Tel Aviv Israel; 2 Sylvan Adams Sports Institute Tel Aviv University Tel Aviv Israel

**Keywords:** motivation, physical activity, social media, Reddit, adherence

## Abstract

**Background:**

Despite the ample benefits of physical activity (PA), many individuals do not meet the minimum PA recommended by health organizations. Structured questionnaires and interviews are commonly used to study why individuals perform PA and their strategies to adhere to PA. However, certain biases are inherent to these tools that limit what can be concluded from their results. Collecting data from social media channels can complement these studies and provide a more comprehensive overview of PA motives and adherence strategies.

**Objective:**

This study aims to investigate motives for engaging in PA, as well as the associated strategies to achieve these goals, as stated by a large number of people on a social media site.

**Methods:**

We searched for users’ responses regarding PA motives and adherence strategies in Reddit forums dedicated to PA and analyzed the data using (1) unsupervised clustering to identify topics from the textual comments and (2) supervised classification to classify the comments into the detected topics. A panel of experts participated in both steps for annotation and validation purposes.

**Results:**

We analyzed 1577 unique user comments (representing 1577 individual users); of those, 1247 were linked to physical appearance (mentioned in 298/1247, 23.9% of the comments) and improving physical (235/1247, 18.9%) and mental health (211/1247, 16.9%), indicating these as the main motivational factors. The main strategies people used to adhere to PA were habit formation (373/1247, 30%), goal setting (173/1247, 13.9%), enjoyable activities (151/1247, 12.1%), socializing (121/1247, 9.7%), using media (111/1247, 8.9%), using different apps to monitor PA (35/1247, 2.8%), and financial commitment (32/1247, 2.5%).

**Conclusions:**

This study presented a novel approach using a language model to investigate why people engage in PA and the strategies they use to adhere to PA using wide-scale, self-disclosed content from popular social media channels.

## Introduction

Despite the established health benefits of physical activity (PA) [1-4], surveys from multiple countries have reported that more than 80% of adolescents and 27% of adults do not meet the World Health Organization guidelines [5-7]. Studying what motivates individuals to perform PA and what strategies assist them in adhering to PA can lead to improvements and developments in interventions aiming to increase PA levels. The most common way to examine people’s motives and adherence strategies to PA is by using standardized questionnaires and structured interviews [8-13]. While these studies using these data collection strategies have clear benefits, they also suffer from several inherent biases that limit what can be concluded based on their results. First, participants in these studies are likely unrepresentative of the population. The observed phenomenon can be attributed to the specific targeting of participants and the resultant self-selection bias, as those who volunteered to participate (ie, volunteer bias) possessed distinct attributes compared with those who declined [14]. Indeed, volunteering is a trait associated with sociodemographic factors, attitudes, and other personality traits of lifestyle behaviors [15,16]. Second, questionnaires and interviews can lead to response bias, given that they can confuse or prompt participants to answer in a particular manner [17,18]. Third, participants may interpret the studies’ purpose or interviewers’ intent and modify their responses to align with this interpretation, a phenomenon known as demand characteristics [19]. These biases might create a misleading picture of reality and lead to misclassification of the factors that motivate individuals to engage and adhere to PA.

One approach that can partially overcome these shortcomings is to collect information concerning motivation and adherence strategies to perform PA from social media channels. Social media allows people to share their experiences and opinions as they wish, mostly unrestrictedly. Moreover, the anonymity afforded by social media may allow for more uninhibited self-disclosure without worrying about potential exposure or various consequences [20-22]. Data gathered in this fashion has inherent biases, including selection bias of social media users proficient in using computers. Nevertheless, the difference in mediums and context allows researchers to complement the traditional approaches based on standardized questionnaires and structured interviews. The increased ease of use in implementing machine learning algorithms on free text has enabled researchers to collect data on social media posts at a massive scale. This method allows for larger sample sizes than traditional research methods and may avoid specific selection and response biases. Yet despite the widespread adoption of this research method within multiple scientific fields, it has been rarely implemented within exercise science, specifically in assessing motivation and adherence strategies for engaging in physical activity.

Of the various social media channels, Reddit is a highly popular website (861 million active users as of 2021) [23] in which users submit text, images, and videos to moderated boards (known as “subreddits”), which are then voted up or down by other users. The websites’ users are primarily from the United States (47.13%) [24], skew young (36% under 30), male (63.8%), and from higher income brackets [25]. Discussions on Reddit are public to anyone (unless specifically designated as private), allowing for passive data collection. Reddit provides an application programming interface (API) for researchers with extensive access to data, making it a valuable source of information. Therefore, this study aims to determine the motivational factors and strategies to both begin and adhere to the PA of Reddit users based on their self-reported data.

## Methods

### Overview

We singled out Reddit from other social media platforms due to its large user base, comment ranking system, mode of posting (questions and answers within subject matter “communities,” self-disclosure), and researcher-friendly API. The process we undertook to identify and extract data from posts on social media was to first decide upon keywords related to PA and motivation based on an initial search to identify relevant communities. Then, the next step was to identify relevant conversations within the selected subreddits. We will now elaborate on each step of this process:

### Reddit Community Identification (Initial Examination, Sorting, and Filtering)

We ran a Google search of Reddit using the keywords “motivation” and “exercise” or “sport.” This search returned 59 communities (ie, subreddits).

We began by excluding subreddits containing fewer than 100,000 users (19 groups) and non-English language groups (2 groups). Following initial examination of the remaining subreddits, we found that they could be broadly divided into 2 categories: “general” (25 groups) and “sports-related” (13 groups). Within these 2 categories, we observed that some subreddits were not topically related to our query (motivation in exercise and sport), but rather, they were geared toward other topics (eg, science, GARMIN, and sports leagues such as the NBA [National Basketball Association] and FIFA [Fédération Internationale de Football Association]) and were therefore excluded (17 and 5 from general and sport-related, respectively). Following further examination of the content of the conversations in the remaining sports-related subreddits (bodybuilding, judo, running), we observed that most of the discussions were of a technical nature (ie, best running strategy, bodybuilding tips) and were therefore excluded. We then selected for further analysis the 3 most popular (based on active user activity) subreddits from the categories identified: PA/sports-related (/r/bodyweightfitness, /r/crossfit, and /r/Fitness) and general advice (/r/askMen, /r/askWomen, and /r/askReddit).

### Comment Identification

#### Comment Search and Initial Data Extraction

We searched the selected subreddits using the Reddit API for conversations containing the terms “motivation,” “exercise,” or “sport.” From each of these discussions, we extracted the title, comments, and total “points” (a site metric that captures how many people liked each comment) and unique author identification number of individual comments.

#### Comment Filtration

In cases where a specific user made more than 1 comment across all the conversations captured by our query, a single comment was chosen randomly. This approach reduces biases of users who posted multiple comments, which can give more weight to their answers and bias the dataset toward prolific commenters rather than unique comments. Based on previous research in this field [26,27], comments containing only emojis, URLs, non-English languages, or one-word responses were also excluded.

### Data Analysis Procedure

**Figure 1:**
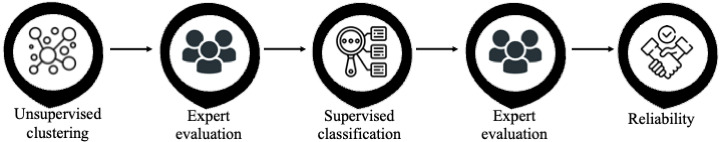
Overview of the main steps of the data analysis pipeline.

To analyze the dataset generated by our data collection and filtration procedure, we used the following analysis pipeline that included 5 steps, as presented in Figure 1. Following the established steps during the development and evaluation of machine learning and artificial intelligence models, it is essential to ensure the accountability and reliability of these models [28,29]. All data analysis was carried out in Python version 3.9.13 (Python Software Foundation) using the fuzzy-c-mean 1.7.2 (FCM), pandas 1.5.3, NumPy 1.26.4, and umap-learn-0.5.6 packages.

### Step 1: Exploration and Initial Clustering

The first step aimed to explore the data and create initial clusters of topics (ie, motivations for engaging in PA) that appear in the text. First, we created embeddings, or vector representations, of each sentence to use for the clustering. Specifically, we encoded using the Sentence Bidirectional Encoder Representations from Transformers language model. We then reduced the high dimensionality of the embeddings using Uniform Manifold Approximation and Projection for Dimension Reduction, introduced by McInnes et al [30], implemented in umap-learn. Finally, we used a clustering algorithm FCM, with FCM implementation [31] to find groups within the data with the most similarity in the same cluster and the most dissimilarity between different clusters (this approach was chosen due to the observation that our data contains examples that discuss several topics). Specifically, this step allows finding groups of similar comments (which are converted to numerical data points) in the data by calculating the distance between data points (see subheading A.1 in Multimedia Appendix 1).

### Step 2: Expert Tuning and Validation

To verify the clusters’ topics, one of the co-authors (IH), with expertise in exercise science and motivation, reviewed several samples and proposed a topic title for each cluster. Then, 30 samples were randomly selected from each cluster, and 2 additional reviewers with similar expertise independently decided whether each sample matched the proposed cluster topic. Note that a sample could match several topics simultaneously. If both reviewers agreed on the topics, the sample was added to a pool of corrected annotated samples. Thus, at the end of this step, we had a high-quality representation of the different topics in the dataset.

### Step 3: Supervised Text Classification

We classified the remaining nonreviewed comments into the topics identified in steps 1 and 2. This procedure’s goal was to automatically classify the textual comments into one or more defined topics (Multimedia Appendix 2 [32-35]).

### Step 4: Expert Evaluation

**Figure 2:**
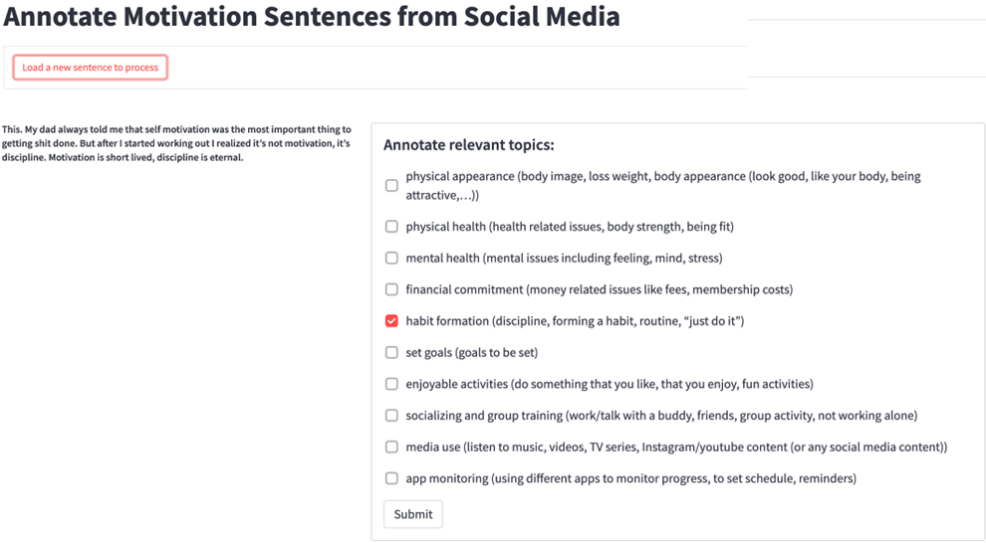
Annotation user interface used by subject matter experts.

We implemented an additional round of expert evaluation of the classified comments. To minimize the risk of researcher bias, we recruited 5 graduate-level exercise physiology students rather than using the original 3 experts. We randomly sampled 30 instances from our data (not used in the first round of evaluation) and asked each expert to evaluate the classified data. Each expert was presented with a dedicated user interface (Figure 2). Each sample was displayed along with the possible topics (multiple choices were allowed) and a field to add comments using free text. Before beginning the annotation task, the reviewers (5 graduate-level exercise physiology students) underwent a familiarization procedure by reading the instructions and practicing on 3 fictitious examples, similar to comments from the dataset regarding topics and average comment length. Once the annotation task was completed, we aggregated the results and evaluated the quality of the classifier and the interrater agreement.

### Step 5: Reliability

To ensure reliable findings, the quality of the supervised classifier that identifies motivational factors and strategies was evaluated using both automatic metrics (precision, recall, and *F*_1_-score) and an expert evaluation panel, as mentioned above in step 4. For the human evaluation, we used Fliess κ interrater reliability [36].

### Ethical Considerations

This research did not require approval from the Tel Aviv University Ethics Committee as it was based on publicly accessible information that is legally protected and does not necessitate an ethics review. This position was confirmed by the head of the university’s ethics committee. From a privacy perspective, we used data from Reddit, a platform where most discussions are publicly accessible to anyone, with or without a Reddit account (except for private subreddits). In addition, Reddit offers an extensive API that allows access to much of the platform’s information. Strictly adhering to the rules and ethical guidelines of each subreddit, we only collected data from subreddits that explicitly allow research activities. Furthermore, to protect user privacy, we only recorded the comment ID without including any user information or the content of the comment itself, ensuring no personally identifiable information or data that could lead to reidentification was collected.

## Results

### Data Collection

We collected 2387 comments from 70 discussions published between 2017 to 2021. After completing the data filtration procedure, 1850 samples remained, submitted by 1850 unique users: 227 from /r/bodyweightfitness, 152 from /r/Fitness, 220 from /r/crossfit, 338 from /r/askReddit, 479 from /r/askMen, and 434 from /r/askWomen. Links and examples of the discussions can be found under subheading A.3 in Multimedia Appendix 1.

### Primary Outcome

Our model classified the comments into 10 distinct topics, which we observed could be divided into 2 broad categories: motivational factors for initiating PA (motivations) and strategies or suggestions on staying motivated and adhering to PA (strategies), for a total of 1740 observations of identified topics, over 1247 comments. The motivations for participating in PA (frequency of appearance) were physical appearance (298/1247, 23.9%), physical health (235/1247, 18.9%), and mental health (211/1247, 16.9%). The main strategies people use to adhere to PA were habit formation (373/1247, 30%), goal setting (173/1247, 13.9%), choosing enjoyable activities (151/1247, 12.1%), socializing (121/1247, 9.7%), using media (111/1247, 8.9%), using different apps to monitor PA (35/1247, 2.8%), and financial commitment (32/1247, 2.5%). Representative examples per topic are presented in Table 1, with their sums and proportions in Table 2. For a breakdown of topic frequency by subreddit, see subheading A.4 and Figure S1 in Multimedia Appendix 1. The reliability and accuracy metrics of the topic allocation by the model were high. We observed substantial agreement between raters with a Fliess κ of 0.621. The subsequent automatic evaluation of the model’s precision, recall, and *F*_1_-scores were 0.86 for all metrics. For a detailed breakdown of this process, see subheading A.4 in Multimedia Appendix 1.

**Table 1:** Topics and representative comments examples.

Topic		Example comments
**Motivational factors for initiating physical activity**		
	***Physical appearance***	
		*Stand in front of the mirror. Naked.*
		*the ass of my dreams*.
		*Buy a scale. Understanding exactly how much your weight is changing has helped me.*
	***Physical health***	
		*If I don’t my fibromyalgia pain will get worse. That’s a pretty daunting thought. I can just barely make it through the day as it is*.
		*I don’t want to die of something that could have easily been prevented by having a basic level of fitness. It’s really the only reason I work out. I’m pretty lazy*.
		*Good to stay healthy and live a long life*.
	***Mental health***	
		*Exercise makes me happy, improves my mood and helps me be more productive overall*.
		*It helps my mental health*.
		*i try to do three to four, make two a priority found that my mental health (specifically anxiety) started to slide if i did less than two a week. There’s a huge difference in my anxiety levels when my workouts are regular*.
**Strategies or suggestions on staying motivated and adhering to physical activity**		
	***Habit formation***	
		*by starting-the more you do it, the better you’ll feel, etc. it becomes a habit/vicious cycle*.
		*Motivation depends on emotions which are unstable. Discipline is the ability to maintain a habit even when the motivation isn’t there*.
		*It’s addictive. It’s difficult to start but once you get going you can’t get enough*.
	***Goal setting***	
		*You really have to want it. If you don’t care to change or don’t have any numerical goals, you won’t last. Set some goals and really strive to meet them*.
		*I have goals to make a high level rugby side and i need to be fit and strong to get there*.
		*I suggest picking a goal. Run a 5k, 10k, marathon, whatever. Do a one-legged squat, one-armed push-up, ten pull-ups, whatever. Picking goals and tracking my progress towards them has helped me*.
	***Enjoyable activities***	
		*This exactly. I hate going on runs or going to lift weights at the gym. I can’t get motivated to do that. But if I take a spin class or kickboxing or muscle endurance class I I love it. It’s all about finding something you like*.
		*For me, having a sport/game/activity that I enjoy is my motivation. I work out in order to be able to have more fun in that sport/game/activity. With more strength and flexibility, I am more freely and thoroughly able to express myself through movement. I work out so that my play is even more fun*!
		*Do something fun for exercise. For example, I love basketball so I play basketball as an exercise but I still enjoy it and it doesn’t even seem like work*.
	***Socializing***	
		*I enjoy being around the people at my gym. They make showing up everyday fun*.
		*My foolproof method: get a gym buddy. It’s hard to skip when you know someone is waiting for you at the gym*.
		*I have a friend from my gym meet me on zoom and we work out together.it Helps so much*.
	***Media use***	
		*Music and proper clothes. throw on an exercise bra and shorts, plus some salsa music and i can work out for hours. Music is just good for energy*.
		*Play intense music. That’s all it takes for me*.
		*Mhm my favourite workout YouTuber is Lily Sabri. She has many 5-7min videos, so after like a half an hour workout or live stream workout if you still feel like you have a bit more energy you can get one more work out quickly*.
	***App monitoring***	
		*Every day. I got a good streak going on my fitbit from March and I’m desperate not to break it*.
		*I’m using one of the Couch to 5K apps that basically tells you exactly what to do, tracks your progress etc*.
		*Use the IIFYM calculator to figure out how much to eat for your goals and be strict on tracking with myfitnesspal app. 100% you will see great results and never have to seek motivation outside again*.
	***Financial commitment***	
		*Think about how much your paying, I’m NOT wasting $150 per month, get your ass to the gym*.
		*The cost*.
		*I think about how much I pay per month*.

**Table 2:** Total number of comments per topic (and percentage) as classified by the model.

Topic			Comments (n=1247), n (%)
**Motivational factors for initiating physical activity**			
	Physical appearance	298 (23.9)	
	Physical health	235 (18.9)	
	Mental health	211 (16.9)	
**Strategies or suggestions for staying motivated and adhering to physical activity**			
	Habit formation	373 (30)	
	Goal setting	173 (13.9)	
	Enjoyable activities	151 (12.1)	
	Socializing	121 (9.7)	
	Media use	111 (8.9)	
	App monitoring	35 (2.8)	
	Financial commitment	32 (2.5)	

### Additional Exploratory Analysis

#### Time Period

The lockdowns and social distancing behaviors associated with the COVID-19 pandemic were known to have caused significant changes in population PA levels [37]. Therefore, we examined the frequency distribution of the 10 identified topics during 2 periods of time: before the pandemic, defined as the period up to date that a public health emergency was declared in the United States (January 1, 2017, to January 31, 2020; 940 comments) and during the pandemic (February 1, 2020, to January 31, 2021; 307 comments). Specifically, to calculate the topic frequencies before the pandemic, we summed the number of comments per topic and divided it by the total number of comments during this period (940). A similar calculation was done for the duration of the pandemic period (307). We observed statistically significant differences between the 2 time periods (using a 2-sample *z* test for proportions) in all 3 motivations for engaging in PA (physical appearance: *P*=.01; physical health: *P*<.001; mental health: *P*<.001) and in 2 strategies (enjoyable activities: *P*=.04; financial commitment: *P*=.01), as depicted in Table 3.

**Table 3:** Overview of the motivations and strategies before and during the COVID-19 pandemic (2-sample z test for proportions).

Topic	Before COVID-19 pandemic	During COVID-19 pandemic
Physical appearance^a^	0.26	0.19
Physical health^a^	0.17	0.25
Mental health^a^	0.15	0.24
Habit formation	0.30	0.29
Set goals	0.14	0.14
Enjoyable activities^a^	0.13	0.09
Socializing and group training	0.11	0.07
Media use	0.09	0.08
App monitoring	0.03	0.03
Financial commitment^a^	0.03	0.01

^a^*P*<.05.

#### Topic Co-Occurrence

**Figure 3:**
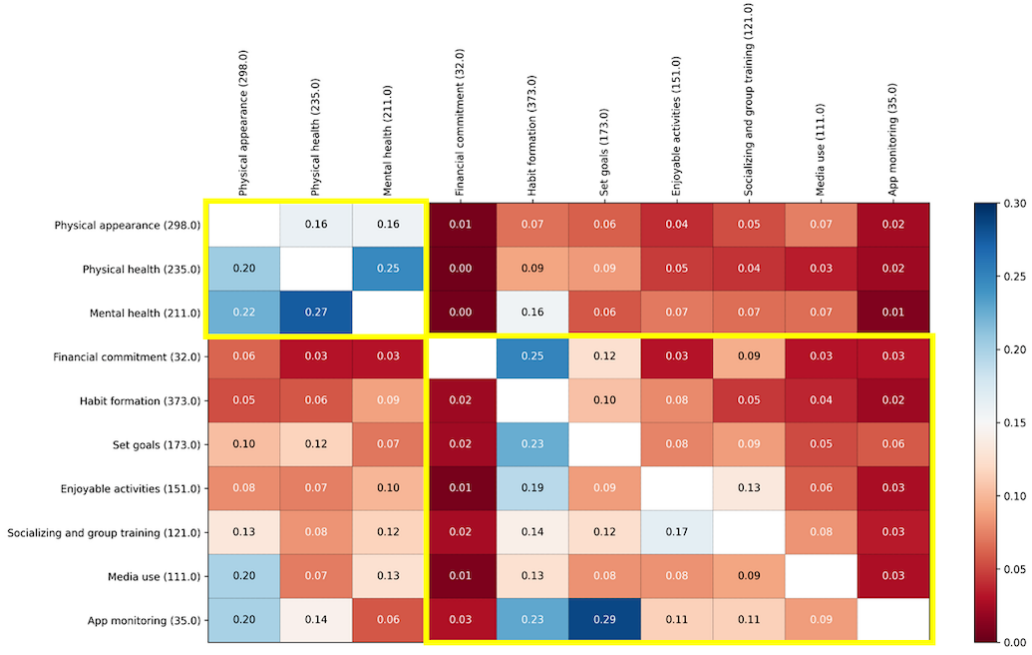
Topic co-occurrences probabilities. Each cell is the probability of a row’s topic appearing together with a column’s topic (calculated as the number of co-occurrences divided by the total appearances of the row topic, that is, the number in parentheses). The cells within the 2 yellow outlines represent co-occurrence probabilities of motivations to motivations and strategies to strategies.

We elected to examine patterns of topics mentioned in users’ comments, that is, the probability of a Reddit user’s comment containing combinations of topics (2 motivations or strategies). Out of 1740 unique observations of identified topics, 1278 incidences of topic co-occurrence were identified. Figure 3 presents a heat map of the co-occurrence probabilities, where each cell in the matrix presents the probability of a column’s topic co-occurring with a row’s topic*,* normalized by the total appearances of the row (as presented in Table 2).

For example, given a comment labeled as discussing the topic of “physical health,” the probability of that comment also discussing “mental health” was 0.25. We observed that the co-occurrences with the highest probabilities were combinations of “motivational factors/motivational factors” rather than “motivational factors/strategy.”

#### Topic Importance

**Figure 4:**
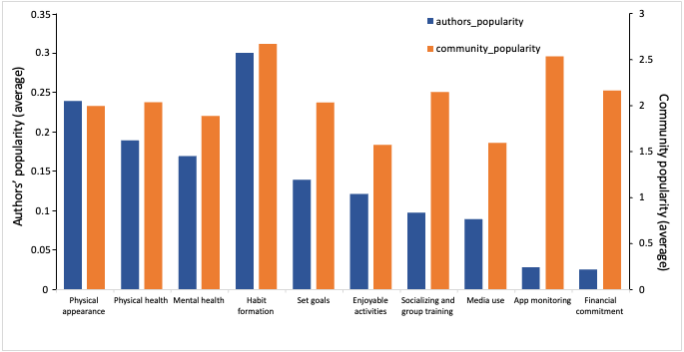
Importance analysis per topic by the popularity of the authors and the community. Author popularity (per topic) = number of authors who wrote about the topic ÷ total number of authors. Community popularity (per topic) = total scores given to the topic by the community ÷ number of comments on the topic.

Some of the 10 identified topics may be considered more “important” than others by the users of the various Reddit communities studied here. The simplest method of defining importance would be by the frequency in which it appears in the dataset, as shown in Table 2. This metric can alternatively be described as the topic’s popularity among comment authors (recall that the data filtration process allowed the inclusion of only 1 comment per author). As the Reddit API provided us with additional contextual data for each comment (comment “points”), we added the metric of topic importance using popularity “points”—how well those comments, and hence, topics, were received or recognized. Each Reddit comment is associated with points. The community members give points representing reaction agreement (or likes) on a specific comment. Thus, the more points a comment has, the more well-received a comment is by others. We used this point system as a proxy of topic popularity or importance. Specifically, we collected the total number of scores per topic and averaged them by the number of topic comments. We then compared the “popularity” of the 10 topics using the 2 measures proposed above. Figure 4 compares these 2 metrics, average authors versus average community importance, by topic.

## Discussion

### Principal Findings

Our objective in this study was to explore motivational and adherence strategies for engaging in PA by using an unsupervised, multistep clustering technique to gather and analyze comments from Reddit forums. Our model classified the comments into 10 distinct topics, which we further categorized into 2 themes: motivations for initiating PA and strategies for maintaining motivation and adherence to PA. The main motives for individuals to engage in PA was their physical appearance (298/1247, 23.9%) and enhancing physical (235/1247, 18.9%) and mental (211/1247, 16.9%) health. The most prevalent strategy individuals used to adhere to PA was habit formation (373/1247, 30%), followed by goal setting (173/1247, 13.9%), enjoyable activities (151/1247, 12.1%), socializing (121/1247, 9.7%), using media (111/1247, 8.9%), using PA monitoring apps (35/1247, 2.8%), and financial commitment (32/1247, 2.5%). In addition, the frequency with which certain topics were mentioned together presented with certain patterns (Figure 3). For example, mental or physical health were often mentioned together with physical appearance (385/1247, 22% and 348/1247, 20%, respectively), and app monitoring or media use were often mentioned together with physical appearance (348/1247, 20% and 348/1247, 20%, respectively). These results shed some light on the multifactorial motivations underlying PA and hint at certain strategies individuals may gravitate to, depending on their primary motivational factor to initiate PA.

Comparing and contrasting our results to other studies using more traditional approaches presents challenges due to the different methodologies implemented. Nevertheless, our results are partly aligned with studies assessing PA motivation through questionnaires based on the Self-Determination Theory (SDT) framework, such as the Exercise Motivation Measurement scale (EMI-2) [38]. The EMI-2 takes the SDT spectrum of motivations and further divides them into subscales based on various topics (eg, “ill health avoidance,” “social recognition”). For example, we identified 3 motivations for participating in PA (physical and mental health and physical appearance) consistent with the subscales identified in such studies. This supports our model’s construct validity and SDT-based questionnaires’ external validity, as our unprompted findings closely mirror several subscales of the established questionnaires. Yet, our results are also misaligned with some of the literature. Regarding the EMI-2 scale, our model failed to identify specific “personal” and “social” motivation-related subscales, such as challenge, enjoyment, social recognition, and affiliation. We speculate that this misalignment stems from the unique characteristics of Reddit (public, online, anonymous posts) and the characteristics of the population that tends to use it (younger, male, western internet users) who may feel less inclined to discuss more personal motivations openly. We make this assumption with the caveat that we do not know if the demographic attributes of the subreddits we sampled truly mirror those of Reddit at large.

Our results seem to better align methodologically with qualitative studies based on semistructured interviews, showing a similar distribution of motivations in health-related topics [12,39,40]. For example, Ashton et al [40] identified 4 themes for motivators for PA among young adult men (a similar demographic to the average Reddit user): physical and mental health, physical appearance, social inclusion, and sport or performance. Here, we see that, similar to the quantitative literature, our model aligned with the health-related categories but did not identify social and personal motivational themes, such as skill development, support networks, community, relationships, and competitiveness. Direct comparison to qualitative studies on the topic is also challenging, given that they generally use thematic analysis as their research tool of choice. In contrast, we quantified the prevalence of topics within a large sample, as presented in this study, in a manner similar to qualitative content analysis. Furthermore, the process we used for assigning names to topic clusters, performed by a single reviewer without a formalized content analysis process, might have led to the misclassification of specific categories. Yet, a careful review of the text categorized by our model reveals a clear distinction between discussions on “how” to motivate oneself to engage in or adhere to PA versus the underlying “why” behind engaging in PA. We acknowledge this as a consideration for future research using wide-scale, self-disclosed content and recommend implementing established methods from the field of qualitative content analysis [41].

Several limitations of this study are worthy of discussion. First, the lack of data on the demographic attributes of the comment authors beyond platform-wide industry reports [25] limits our ability to associate the observed motivations and strategies with specific populations (ie, gender and age groups). Furthermore, although there are certain natural language processing techniques that can attempt to infer a user’s gender based on previous comments written by the user, in our case, they were not able to assign gender to a large portion of our sample and thus excluded from the final analysis. Second, it is important to note that the questions that our analysis can answer are limited in scope as it was meant to be a proof-of-concept. The relatively simple queries we constructed were not designed to identify posts containing more detailed information, such as whether users were meeting minimal exercise standards, the location of exercise, or the results of implementing the identified strategies. In addition, all self-disclosed information, especially internet-based and pseudo-anonymous, must be interpreted with caution as the difference between disclosure and reality may be significant. Third, the “data-driven research” process used here allowed for substantial researcher degrees of freedom during the initial data selection and filtration process, potentially leading to selection bias, among others. Future studies could implement a more formalized process for topic classification and setting inclusion or exclusion criteria, enabling the answering of more complex questions and further refining our approach. Despite these limitations, incorporating human experts in auditing the automated results throughout the different stages of the analysis process is an essential strength of our methodology.

### Conclusion

Our study introduces a novel usage of language models to harness the content shared on social media platforms to investigate the motivations and adherence strategies of individuals engaging in PA. Our findings further support the established literature and identified self-reported strategies implemented by this population for initiating and sustaining PA. The insights gained here provide valuable information and research tools for better understanding PA motives and adherence strategies. By leveraging self-disclosed data in an unprompted, nonresearch context on a large scale, we have expanded possibilities for research and gained more profound insights into people’s thoughts, feelings, and opinions. This method has been successfully applied to various fields, and its application to PA and motivation represents a significant contribution. This study provides proof-of-concept for future investigations and opens new avenues for using language models in social media channels to study PA behavior and motivation.

## Appendix

Multimedia Appendix 1 Topic identification using clustering.

Multimedia Appendix 2 Example of the JSON file format.

## Abbreviations

API: application programming interface
EMI-2: Exercise Motivation Measurement scale
FCM: fuzzy-c-mean
PA: physical activity
SDT: Self-Determination Theory

## Data Availability

The datasets generated during and/or analyzed during this study are available in the GitHub repository [42]. The JSON file includes the comment_id as “id” (as the textual content cannot be shared by the agreement rules); using the Reddit API, other researchers will be able to fully reconstruct the dataset. An example of the JSON file format is mentioned in Multimedia Appendix 2. Examples of the textual content can be found in Table 1.
