# Supplementary material for: Analysis of Reddit Discussions on Motivational Factors for Physical Activity: Cross-Sectional Study
Source: J Med Internet Res. 2025 Jan 13;27:e54489. doi: 10.2196/54489 (PMC11773284; doi:10.2196/54489)
Supplement: Multimedia Appendix 1 [file jmir_v27i1e54489_app1.docx]

**A.1 Topic identification using clustering:** This step aimed to explore the data and create initial clusters of reasons for motivation that appear in the text using unsupervised text clustering. First, it was necessary to represent our text data numerically. A common approach is to create embeddings, or vector representations, of each sentence to use for the clustering. Specifically, we encoded using Sentence-BERT (SBERT) [‎38]. Sentence embeddings have high dimensionality (>500 features each). Thus, we use Uniform Manifold Approximation and Projection for Dimension Reduction (UMAP), introduced by McInnes et al [‎39]. UMAP is fast and preserves the global structure of the data (using umap-learn package [‎30]). Given this input (embedded and dimension reduce),we used a fuzzy c-means clustering algorithm (FCM) following the approach presented by Yang et al [‎40], using the fuzzy-c-mean package [‎31].

**A.2 Comments Classification** **Method:** Given the topics and small high-quality human labeled data, we aimed to classify the rest of the data into the topics by fine-tuning a BERT [‎41] model on the task of NLI (natural language inference). An NLI model is asked to identify some relationship between 2 sentences: a hypothesis and a premise. If the premise and hypothesis are compatible, this pair represents an entailment; conversely, if they are incompatible, the pair would be labeled as a contradiction. In our annotated data, the premise was the user comment, the hypothesis was the topic (along with a relevant prompt), and the label was “entailment”. We created 2 prompts, one for the topics belonging to the motivational factors “The motivation to exercise in this sentence is”. The other prompt was for the topics of the techniques “The strategy to keep exercise in this sentence is”. Table 4 shows 2 examples of instances from our data.

| Premise: “Feeling mentally better after my training is my main motivation. If I don’t work out I feel like @@@@.”  Hypothesis: “The motivation to exercise in this sentence is mental health”.  Label: Entailment |
| --- |
| Premise: “My trick is really simple: find an activity I like doing.”  Hypothesis: The strategy to keep exercise in this sentence is enjoyable activities.”  Label: Entailment |

**Table 4. Example of 2 samples represented as NLI instances.**

**A.3 Examples of Discussion topics on subreddits**. Table 5 shows examples of relevant discussions topics per subreddit. As can be observed, a discussion consisting of a title (question) that was posed by a user in the subreddit community. For example, the question in the AskMen subreddit’s first thread is “What motivates you to work out?”. When entering a discussion (by clicking on its title), all the comments of the discussion will be shown.

| Subreddit | Examples |
| --- | --- |
| AskMen | what_gives_you_the_motivation_to_workout  men_how_do_you_get_yourself_motivated_to_workout  how_do_you_guys_get_motivated_enough_to_workout  how_do_yall_have_the_motivation_to_exercise |
| AskWomen | what_motivates_you_to_exercise  what_has_motivated_you_personally_to_exercise_and  do_you_workout_if_so_how_do_you_stay_motivated  how_do_you_motivate_yourself_to_work_outgo_to_the |
| AskReddit | fit_people_of_reddit_how_do_you_getstay_motivated  how_do_you_motivate_yourself_to_exercise  reddit_whats_your_motivation_to_exercise  reddit_how_do_you_motivate_yourself_to_exercise |
| Bodyweightfitness | how_do_you_motivate_yourself_to_workout  how_can_i_get_motivated_to_workout  im_lazy_how_do_you_stay_motivated_to_work_out  what_do_you_guys_look_to_for_motivation |
| Fitness | when_you_lose_that_fire_inside_of_you  lost_motivation_to_train  how_to_find_motivation_to_work_out_after_long  i_feel_like_i_have_lost_motivation_to_go_to_the |
| Crossfit | has_anyone_else_lost_their_motivation_to_work_out  help_lost_gains_lost_motivation  how_do_you_get_lost_motivation_back  keeping_motivation_up |

**Table 5. Examples of motivation-related discussions in different communities.**

**A.4 Detailed results of the pipeline stages.**

Following *step 1* of our analysis pipeline – unsupervised text clustering – and the initial expert tuning stage of the pipeline (*step 2*) - our model identified ten clusters of topics (Table 2). The distribution of agreed-upon comments following the initial expert tuning stage of the pipeline (*step 2*) is presented in Table 6 (273 comments).

Using the 273 annotated examples generated by the two domain experts in *step 2*, we implemented *step 3* of the analysis pipeline by training a classifier – (further details on the training are in Multimedia Appendix A.2). We then classified the remaining comments (those that were used for the initial annotation). In total, 1577 comments were evaluated by the model, out of which only 1247 comments were classified into the topics, and 330 comments were not matched with any of the topics (e.g., “Work out ten times over the next 14 days. That MIGHT motivate you.” or “Go get em’.”, did not match with any of our topics.)

Next, we implemented *step 4* – a second round of expert evaluation. We randomly extracted 30 samples from the classified comments, which underwent evaluation by the five additional experts. We aggregated the results and relied on a majority vote. For the topic verification, the inter-rater reliability measured by Fliess Kappa was 0.621, which indicates substantial agreement [‎30]. Table 7 summarizes the accuracy per topic by majority votes. In *step 5*, automatic evaluation of the model - precision, recall, and F1 scores were calculated with 0.86 for all metrics.

|  | Topic | #Comments |
| --- | --- | --- |
| *Motivational factors for initiating physical activity* | physical appearance | 62 |
|  | physical health | 43 |
|  | mental health | 68 |
| *Strategies or suggestions for staying motivated and adhering to physical activity* | habit formation | 74 |
|  | goal setting | 32 |
|  | enjoyable activities | 19 |
|  | socializing | 40 |
|  | media use | 27 |
|  | app monitoring | 3 |
|  | financial commitment | 26 |

**Table 6. Total number of comments per topic as agreed by the annotators.**

|  | Topic | #Comments | *F_1_* score |
| --- | --- | --- | --- |
| *Motivational factors for initiating physical activity* | physical appearance | 13 | 0.87 |
|  | physical health | 6 | 1 |
|  | mental health | 7 | 0.77 |
| *Strategies or suggestions for staying motivated and adhering to physical activity* | habit formation | 8 | 1 |
|  | goal setting | 5 | 0.93 |
|  | enjoyable activities | 7 | 0.8 |
|  | socializing | 5 | 0.75 |
|  | media use | 2 | 1 |
|  | app monitoring | 1 | 1 |
|  | financial commitment | 2 | 0.66 |

**Table 7. Model *F_1_* score for 30 samples annotated by 5 experts.**

**
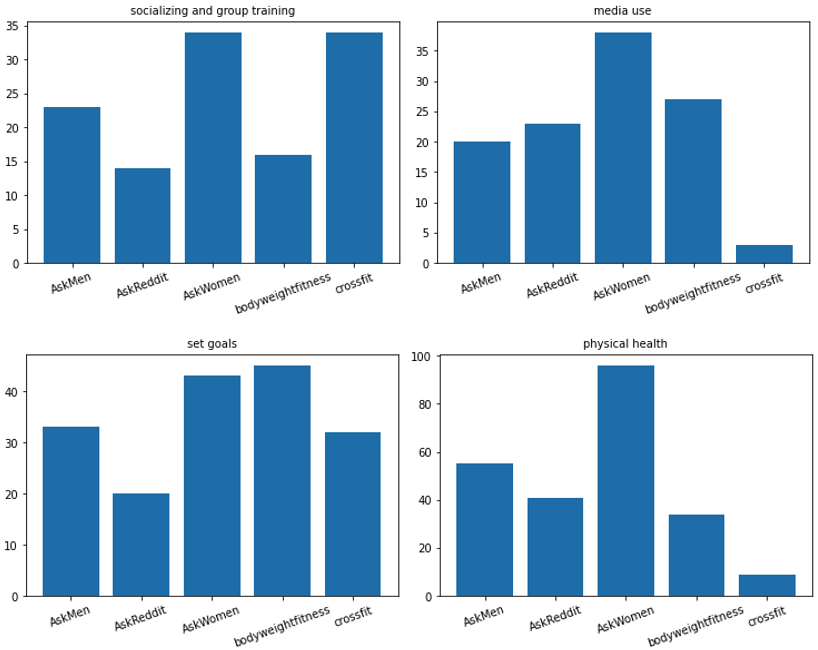
**

**
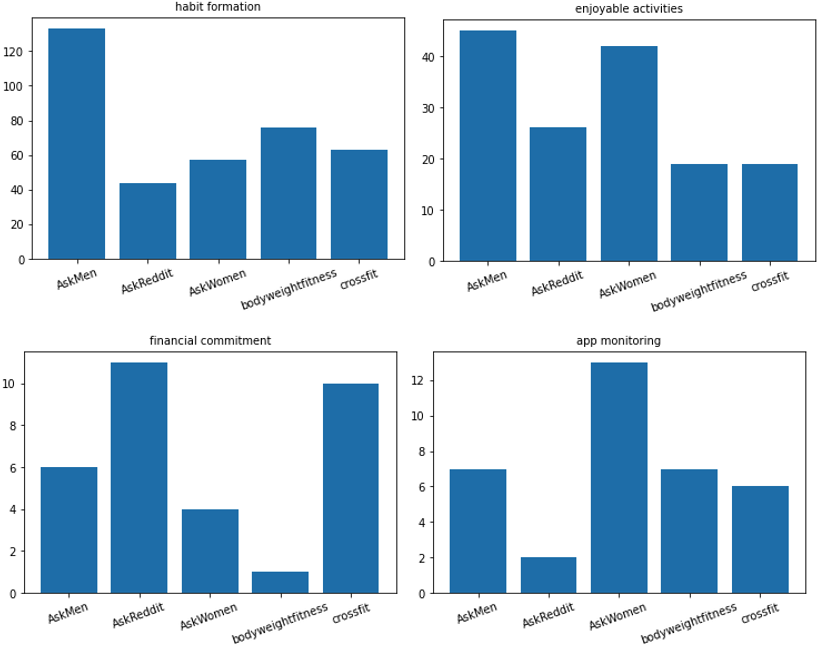
** **
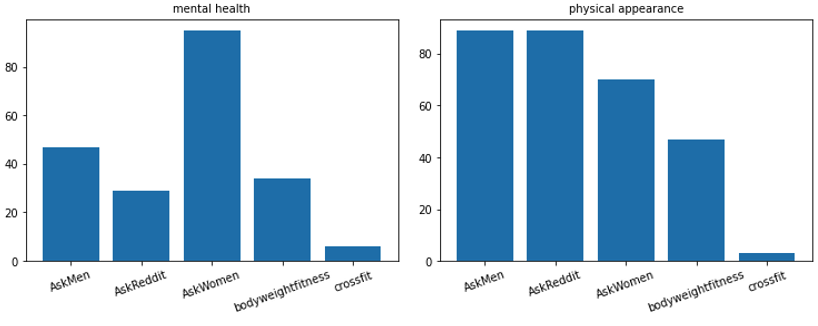
**

**Figure S1. Topic frequency per subreddit.**
