# Supplementary material for: Analysis of Reddit Discussions on Motivational Factors for Physical Activity: Cross-Sectional Study
Source: J Med Internet Res. 2025 Jan 13;27:e54489. doi: 10.2196/54489 (PMC11773284; doi:10.2196/54489)
Supplement: Multimedia Appendix 2 [file jmir_v27i1e54489_app2.docx]

**Example:**

[

{

"sample_id": 729,

"id": "r/AskReddit/comments/t3_3h7kg3/comment/cu5com6/",

"question": "r.AskMen.guys_who_work_out_regularly_how_do_you_motivate",

"labels": [

"physical appearance"

]

},

{

"sample_id": 379,

"id": "r/AskReddit/comments/t3_3h7kg3/comment/cu5j5eq/",

"question": "r.AskReddit.serious_reddit_how_do_you_find_the_motivation_to",

"labels": [

"physical appearance"

]

},

{

"sample_id": 396,

"id": "r/AskReddit/comments/t3_3h7kg3/comment/cu5com6/",

"question": "r.AskReddit.serious_reddit_how_do_you_find_the_motivation_to",

"labels": [

"set goals"

]

},

]
